# Electricity in Cases of Poisoning by Laudanum

**Published:** 1844-07-27

**Authors:** 


### ELECTRICITY IN CASES OF POISONING BY LAUDANUM.

Mr. Corfe, of the Middlesex Hospital, has related an instance of the good effects of electricity under these circumstances. A man was admitted, having taking an ounce and a half of laudanum on the preceding evening, six hours previously. "In the first instance I ordered the administration of the stomach-pump, at which period, to all appearances, he was a lifeless corpse; the pupils were contracted to a pin-hole in size; the pulse was intermitting, and not more than 40; the respirations convulsively performed at intervals of half a minute; the face livid, and the extremities bluish and cold. After the stomach had been relieved of its contents, green tea, with ammonia, was injected therein; flagellation with thin splints and wet towels, the cold douche, turpentine stupes and sinapisms to his calves and abdomen, were applied in succession, without the least improvement in his condition. The bladder was relieved of six or eight ounces of light-coloured urine by the catheter. I then thought of a most powerful remedy, which was attended with extraordinary success; I allude to the electro-magnetic battery, conjointly with electricity, which was set to work upon him soon after four o'clock. The pulse became more steady, firm, and frequent; the respirations more indicative of resuscitation. Our powerful electrical machine was now got into full play before a large fire, and the jar filled, when some brilliant sparks and strong shocks were occasionally passed through his head, spine, thorax, and abdomen."—*Med. Chir. Rev. from the Lancet*, Jan. 27, 1844.

The result of this was, that the man opened his eyes, and his mouth too, abusing the operators for a pack of rascals who were "trying specimens" on him. But incomparably the most satisfactory effect was produced by giving him a shock on the tip of his nose. To use a phrase of the ring, he *rallied* wonderfully under this—a hint worth taking.

